# Cerebrospinal fluid and plasma biomarkers in individuals at risk for genetic prion disease

**DOI:** 10.1186/s12916-020-01608-8

**Published:** 2020-06-18

**Authors:** Sonia M. Vallabh, Eric Vallabh Minikel, Victoria J. Williams, Becky C. Carlyle, Alison J. McManus, Chase D. Wennick, Anna Bolling, Bianca A. Trombetta, David Urick, Chloe K. Nobuhara, Jessica Gerber, Holly Duddy, Ingolf Lachmann, Christiane Stehmann, Steven J. Collins, Kaj Blennow, Henrik Zetterberg, Steven E. Arnold

**Affiliations:** 1 Henry and Allison McCance Center for Brain Health, Massachusetts General Hospital, Boston, MA 02114 USA; 2 Department of Neurology, Massachusetts General Hospital, Boston, MA 02114 USA; 3 Stanley Center for Psychiatric Research, Broad Institute of MIT and Harvard, 415 Main St., Cambridge, MA 02142 USA; 4 Prion Alliance, Cambridge, MA 02139 USA; 5 AJ Roboscreen GmbH, 04129 Leipzig, Germany; 6 Australian National CJD Registry, University of Melbourne, Parkville, 3010 Australia; 7 Department of Psychiatry and Neurochemistry, the Sahlgrenska Academy at the University of Gothenburg, S-431 80 Mölndal, Sweden; 8 Clinical Neurochemistry Laboratory, Sahlgrenska University Hospital, S-431 80 Mölndal, Sweden; 9 UK Dementia Research Institute, University College London, London, WC1N 3BG UK; 10 Department of Molecular Neuroscience, UCL Institute of Neurology, London, WC1N 3BG UK

**Keywords:** Neurodegenerative disease, Cerebrospinal fluid, Biomarkers, Prion, Primary prevention, Clinical trial design, Neurofilament, Total tau, Real-time quaking-induced conversion

## Abstract

**Background:**

Prion disease is neurodegenerative disease that is typically fatal within months of first symptoms. Clinical trials in this rapidly declining symptomatic patient population have proven challenging. Individuals at high lifetime risk for genetic prion disease can be identified decades before symptom onset and provide an opportunity for early therapeutic intervention. However, randomizing pre-symptomatic carriers to a clinical endpoint is not numerically feasible. We therefore launched a cohort study in pre-symptomatic genetic prion disease mutation carriers and controls with the goal of evaluating biomarker endpoints that may enable informative trials in this population.

**Methods:**

We collected cerebrospinal fluid (CSF) and blood from pre-symptomatic individuals with prion protein gene (*PRNP*) mutations (*N* = 27) and matched controls (*N* = 16), in a cohort study at Massachusetts General Hospital. We quantified total prion protein (PrP) and real-time quaking-induced conversion (RT-QuIC) prion seeding activity in CSF and neuronal damage markers total tau (T-tau) and neurofilament light chain (NfL) in CSF and plasma. We compared these markers cross-sectionally, evaluated short-term test-retest reliability over 2–4 months, and conducted a pilot longitudinal study over 10–20 months.

**Results:**

CSF PrP levels were stable on test-retest with a mean coefficient of variation of 7% for both over 2–4 months in *N* = 29 participants and over 10–20 months in *N* = 10 participants. RT-QuIC was negative in 22/23 mutation carriers. The sole individual with positive RT-QuIC seeding activity at two study visits had steady CSF PrP levels and slightly increased tau and NfL concentrations compared with the others, though still within the normal range, and remained asymptomatic 1 year later. T-tau and NfL showed no significant differences between mutation carriers and controls in either CSF or plasma.

**Conclusions:**

CSF PrP will be interpretable as a pharmacodynamic readout for PrP-lowering therapeutics in pre-symptomatic individuals and may serve as an informative surrogate biomarker in this population. In contrast, markers of prion seeding activity and neuronal damage do not reliably cross-sectionally distinguish mutation carriers from controls. Thus, as PrP-lowering therapeutics for prion disease advance, “secondary prevention” based on prodromal pathology may prove challenging; instead, “primary prevention” trials appear to offer a tractable paradigm for trials in pre-symptomatic individuals.

## Background

The well-defined pathobiology of prion disease, with prion protein (PrP) as the sole causal agent [1], has spurred preclinical development of PrP-lowering drugs [2, 3]. The rapid progression of prion disease, which is typically fatal in under a year [4], presents a challenge for drug development, as symptomatic patients may be profoundly debilitated by the time of diagnosis and enrollment [5, 6]. Individuals with mutations in the prion protein (*PRNP*) gene, many of which are highly penetrant [7], may be aware of their risk decades in advance of symptom onset [8], creating an opportunity for early intervention. However, because the unpredictable age of onset precludes randomizing pre-symptomatic individuals to a disease onset endpoint [9], pre-symptomatic trials may instead need to rely on a surrogate biomarker endpoint [10].

“Secondary prevention” trials might recruit pre-symptomatic individuals with prodromal biomarker evidence of disease and aim to stabilize or reverse progression of those markers [11]. While decades-long prodromes are well-documented in slower dementias [12, 13], longitudinal imaging and neurophysiological studies in genetic prion disease have uncovered at most subtle changes in some individuals ~ 1 year before disease onset [14–16]. While there exist a small number of case reports [17–19], to date, no systematic studies have reported on fluid biomarkers in pre-symptomatic prion disease individuals. Neuronal damage markers total tau (T-tau) and neurofilament light (NfL) in both CSF and blood are candidates with dramatic elevation in symptomatic prion disease [17, 20–23] and prodromal elevation in slower dementias [12, 13]. Prion “seeding activity” in CSF measured by real-time quaking-induced conversion (RT-QuIC) is a candidate with excellent diagnostic sensitivity and specificity in symptomatic prion disease [24].

“Primary prevention” trials might recruit pre-symptomatic individuals at known high genetic risk without requirement of prodromal pathology and treat toward a pharmacodynamic biomarker deemed reasonably likely to predict clinical benefit: CSF PrP levels [10]. For reduction in CSF PrP levels to serve as a trial endpoint, mitigation of the dramatic pre-analytical variability observed in historical cohorts [25] will be essential, and because CSF PrP drops in symptomatic disease, biotemporal stability in pre-symptomatic individuals must be assessed.

Here, we recruited and characterized a cohort of pre-symptomatic individuals at risk for genetic prion disease and controls, in order to assess candidate biomarkers for primary and secondary prevention paradigms.

## Methods

### Study design

The study, approved by the Partners Institutional Review Board in April 2017 (protocol #2017P000214), was conceived and designed with the pre-specified, publicly announced (http://broad.io/mghprionstudy) primary goal of evaluating the test-retest stability of CSF PrP concentration in individuals at risk for genetic prion disease. Participants were recruited through IRB-approved advertisements shared by word of mouth and social media by Massachusetts General Hospital, Prion Alliance, and CJD Foundation. Participants included known mutation carriers, individuals at risk (typically 50/50 risk with an affected first-degree relative), and controls including genetic prion disease family members who had already tested negative for a mutation, spouses, and unrelated but demographically matched local controls. Participation required two study visits to Boston and absence of contraindication to lumbar puncture (LP). All participants were cognitively sound and provided written informed consent at the time of study enrollment. This study did not provide predictive genetic testing for genetic prion disease; the research team performed *PRNP* genotyping on de-identified samples for research purposes only. Participant details are provided in Supplementary Methods.

### Lumbar puncture and CSF processing

The lumbar puncture (LP) for CSF collection was performed using a standardized protocol with a 24-G atraumatic Sprotte needle. The time of day for LP was kept consistent across subjects, and 20 mL CSF was collected per subject where possible. Following collection, CSF was handled uniformly according to an established protocol designed to minimize PrP loss to plastic through measures including (i) highly controlled and minimized plastic exposure, (ii) uniform storage in aliquots no smaller than 40 μL, and (iii) addition of 0.03% CHAPS detergent to a subset of CSF to maintain PrP solubility [25]. Samples were then frozen at − 80 °C until analysis at the Broad Institute. CSF aliquots containing 0.03% CHAPS were used for PrP quantification by ELISA; neat CSF aliquots with no additive were used for T-tau ELISA, NfL ELISA, and RT-QuIC. Because some LPs were anomalous or unsuccessful, for some participants, CHAPS CSF, neat CSF, or both were not available. These individuals were excluded from the corresponding analyses.

### Participants

The study was originally designed to recruit 10 mutation carriers and 10 controls, a number expected to be sufficient to characterize test-retest reliability of CSF PrP as a descriptive statistic; enrollment was subsequently expanded as funding allowed. Additional file 1: Figure S1 provides a schematic overview of participation inclusion and exclusion. Data points are omitted from the analysis where missing due to missed visits or unsuccessful LPs. For analyses conducted at MGH (assessments, post-LP survey, CSF total protein), participant data were collected and stored using REDCap [26]. To protect participant privacy, mutations carried by only one individual are grouped as “other,” and the dates of participants’ second and third visits were scrambled by the addition of a normally distributed random variable (mean = 0, standard deviation = 2 weeks or 2 months, for second and third visits respectively).

### Positive control samples from symptomatic prion disease patients

*N* = 26 anonymized pre-mortem cerebrospinal fluid samples from symptomatic sporadic (*N* = 22) and genetic (*N* = 4, all E200K) prion disease cases collected between 2001 and 2017 were shared by the Australian National Creutzfeldt-Jakob Disease Registry (ANCJDR). All cases were autopsy-confirmed as prion disease, except for *N* = 2 genetic cases, which due to the presence of the mutation are highly likely to have been prion disease. Samples received in ~ 0.5 mL aliquots were thawed upon receipt, aliquoted to 100 μL volume, refrozen at − 80 °C, and re-thawed only immediately before analysis. Due to sample volume limitations, not all positive controls were utilized in all assays.

### Assessments of cognitive, neuropsychiatric, motor, and daily functioning

At each study visit, participants completed a battery of cognitive tests and standardized assessments of mood, neuropsychiatric symptoms, motor function, and activities of daily living. The cognitive battery consisted of standard paper and pencil neuropsychological measures including the Montreal Cognitive Assessment (MoCA) [27], Verbal Fluency and Color Word Interference subtests within the Delis-Kaplan Executive Functioning System (D-KEFS) [28], the Grooved Pegboard test [29], Trailmaking Test Parts A and B [30], and the DCTclock test, which is a digitized version of the standard clock drawing test [31]. Participants also completed computerized testing on an iPad consisting of the following subtests from the National Institute of Health (NIH) Toolbox Cognition Battery [32]: (1) Dimensional Change Card Sort, (2) Flanker Inhibitory Control and Attention Test, (3) Picture Sequence Memory Test, (4) List Sorting Working Memory Test, (5) Pattern Comparison Processing Speed Test, (6) Picture Vocabulary Test, (7) Reading Recognition Test, and (8) Auditory Verbal Learning Test (Rey), with supplemental 20-min delayed recall administered after completion of the toolbox. Raw scores obtained from cognitive measures were converted to standardized scores based on population-based normative data published for each test. Administered self-report questionnaires included the Beck Anxiety Inventory (BAI) [33], Beck Depression Inventory (BDI) [34], Measurement of Everyday Cognition-Short Version (ECog-12) [35, 36], Epworth Sleepiness Scale [37], National Prion Monitoring Cohort MRC Scale [38], Motor Aspects of Experiences of Daily Living section of the Movement Disorders Society-Unified Parkinson’s Disease Rating Scale (MDS-UPDRS M-EDL) [39], and the clinician-administered Neuropsychiatric Inventory—Questionnaire (NPI-Q) [40].

### Post-LP survey

Following each LP, participants completed a brief survey that we designed to assess the experience, either on paper or via iPad. They were asked whether they had previously had an LP, and if so, how many. Participants were then asked to mark an X on a 14-cm Likert-type scale to indicate (1) their level of anxiety before the LP procedure and (2) their current feelings at the prospect of a future LP. In both cases, the response was marked on a continuous spectrum bounded by the two extremes of “not anxious at all” and “extremely anxious.” Responses were normalized to the full length of the scale.

### Blood processing

Blood was collected in EDTA tubes, then centrifuged at 1000 rpm to separate plasma for aliquoting into 0.5 mL aliquots. DNA was extracted from whole blood collected on the first study visit. All samples were codified for analysis. Genotypes were used for research purposes only. *PRNP* single nucleotide variants were identified at the Broad Institute Genomics Platform using a custom targeted capture platform developed by Twist Bioscience combined with deep Illumina sequencing. These genotypes were then confirmed, and the presence or absence of octapeptide repeat insertions determined, using a previously described sequencing and gel analysis protocol [41] implemented by Genewiz and/or Quintarabio. Briefly, this analysis uses primers Int5: 5′-TgCATgTTTTCACgATAgTAACgg-3′, DG2: 5′-gCAgTCATTATggCgAACCTTggCTg-3′, and 3′Sal: 5′-gTACTgAggATCCTCCTCATCCCACTATCAggAAgA-3′. A DG2/3′Sal PCR product (wild-type, 804 bp) is subjected to Sanger sequencing while a DG2/Int5 PCR product (wild-type, 464 bp) is run on a 2% agarose gel to identify large indels.

### PrP ELISA

PrP levels were quantified at the Broad Institute using the BetaPrion Human ELISA assay [42], according to the manufacturer’s instructions (AnalytikJena, Leipzig, Germany). As described previously [25], to maintain PrP in solution, CSF samples used for this analysis were handled with close attention to uniformity and were spiked with 0.03% CHAPS detergent immediately after collection. All samples were diluted 1:50 in blocking buffer (0.05% Tween, 5% BSA, 1x PBS) and assayed in duplicate, with samples from the same individual co-located on the same plate to facilitate comparison. Following termination of the colorimetric development reaction, absorbance per well was measured at 450 nm as well as at 620 nm for background subtraction using a FLUOStar Optima absorbance plate reader, then fit to an internal standard curve to generate PrP concentrations in nanograms per milliliter. The operator was blinded to mutation status.

### Total tau (T-tau) ELISA (Broad Institute and University of Gothenburg)

CSF T-tau was measured using the INNOTEST hTau Ag ELISA kit (Fujirebio, Malvern, PA, USA, and Ghent, Belgium) according to the manufacturer’s instructions. Study samples were diluted 1:4; positive control symptomatic prion disease samples were diluted 1:10. All samples were assayed in duplicate with samples from the same individual co-located on the same plate to facilitate comparison. Following termination of the colorimetric development reaction, absorbance per well was measured at 450 nm as well as at 620 nm for background subtraction using a FLUOStar Optima absorbance plate reader, then fit to an internal standard curve. The operator was blinded to mutation status.

### NfL ELISA (Broad Institute)

CSF NfL was measured using the NF-light RUO ELISA (Uman Diagnostics, IBL International, Umea, Sweden) according to the manufacturer’s instructions. Study samples were diluted 1:2; positive control symptomatic prion disease samples were diluted 1:5. All samples were assayed in duplicate with samples from the same individual co-located on the same plate to facilitate comparison. Following termination of the colorimetric development reaction, absorbance per well was measured at 450 nm as well as at 620 nm for background subtraction using a FLUOStar Optima absorbance plate reader, then fit to an internal standard curve. The operator was blinded to mutation status.

### NfL ELISA (University of Gothenburg)

Following CSF collection and processing as described above, uniformly handled 0.5 mL CSF aliquots with no additive were stored at − 80 °C until shipment on dry ice to the University of Gothenburg for analysis. CSF neurofilament light (NfL) was measured using an in-house developed ELISA as previously described [43].

### Simoa analysis of plasma

Following blood processing as described above, 0.5 mL plasma aliquots were stored at − 80 °C until shipment on dry ice to the University of Gothenburg for analysis. Plasma NfL and total tau levels were measured using the single molecule array (Simoa) HD-1 Analyzer (Quanterix, Billerica, MA, USA). For T-tau, the commercially available Tau 2.0 kit was used according to the manufacturer’s instructions (Quanterix). For NfL, a previously described in-house Simoa assay was used [44]. Calibrators were run in duplicate, and obvious outlier calibrator replicates were masked before curve fitting. Samples were run in singlicate with 4-fold dilution. Two quality control samples were run in duplicate at the beginning and end of each run.

### Real-time quaking-induced conversion (RT-QuIC)

The assay was performed according to an established protocol for the detection of prion seeds in CSF [24] that is widely used for the diagnosis of symptomatic prion disease patients. Briefly, truncated recombinant Syrian hamster prion protein (SHaPrP 90-230) was purified from *E. coli* according to established protocols [45], then frozen at − 80 °C following the determination of concentration by NanoDrop. On the day of use, PrP was thawed and centrifuged at 5000×*g* for 5 min at 4 C in a PALL 100-kDa filter tube. Eighty microliters of reaction mix and 20 μL of CSF were combined in each well of a black 96-well plate with a clear bottom (Nunc) with final concentrations as follows: 300 mM NaCl, 10 mM phosphate buffer, 1 mM EDTA, 10 uM thioflavin T, 0.002% SDS, and 1 mg/mL SHaPrP. All samples were loaded in quadruplicate with each plate containing negative control CSF (healthy mutation-negative individuals) and positive control CSF (symptomatic prion disease patients). After sealing (Nalgene Nunc International sealer), plates were incubated in a BMG FLUOstar Optima plate reader at 55 °C for 40 h with continuous cycles of 60 s shaking (700 rpm, double-orbital) and 60 s rest and ThT fluorescence measurements every 45 min (excitation 450 nm, emission 480 nm, bottom read). Following termination of the experiment, fluorescence readings were merged per well to generate kinetic curves, and the threshold for a positive well was set as the mean value of all negative wells plus 10 standard deviations. A sample was considered overall positive if at least two of four replicates crossed this threshold. The operator was blinded to mutation status. For the bank vole prion protein (BvPrP23-230) alternative protocol, RT-QuIC was performed according to the protocol described by Orru et al. [46], with the key modification that 20 μL CSF seed was used per well, rather than 2 μL brain homogenate seed. The final concentrations of reagents in the reaction mix were not changed, and the total reaction volume remained 100 μL per well.

## Results

Mutation carriers and controls were demographically well-matched (Table 1; Additional file 1: Table S1) and performed within established normative ranges across a battery of 20 cognitive, neuropsychological, psychiatric, and motor tests and inventories with no significant differences between groups, supporting the carriers’ pre-symptomatic status (Table 1; Additional file 1: Table S1). No participants developed clinical symptoms of prion disease over the course of the study. The research lumbar puncture (LP) was well-tolerated, and the *N* = 24 participants for whom this was the first LP generally reported lower anxiety about undergoing future LPs than they had felt about the first LP (Additional file 1: Figure S2).

**Table 1:** Demographic overview of study participants. The participant number, age, sex, PRNP genotype, total number of study visits at time of analysis, and scores on two basic assessments of daily and cognitive functioning. Corrected *p* values account for all 20 assessments performed. The “other” category includes four distinct mutations, two of which are of low penetrance and two of which are highly penetrant [7, 9]; to protect participant confidentiality, the exact mutations are not disclosed

			***PRNP*mutation carriers**	**Non-carrier controls**	
***N***			27	16	
**Age at the first visit**			44.2 ± 15.2	44.5 ± 12.7	
**Sex**	Male		10	5	
	Female		17	11	
***PRNP*genotype**	Wild type		0	16	
	E200K		12	0	
	D178N		7	0	
	P102L		4	0	
	Other		4	0	
**Number of completed study visits**	1 visit		7	4	
	2 visits		9	9	
	3 visits		11	3	
		***PRNP*mutation carriers**	**Non-carrier controls**	***p*value**	***p*value (Bonferroni corrected)**
**Assessments**	MRC prion disease rating scale	20.0 ± 0.0	20.0 ± 0.0	1	1
	Montreal cognitive assessment	27.7 ± 1.6	28.5 ± 1.7	0.20	1

**Fig. 1:**
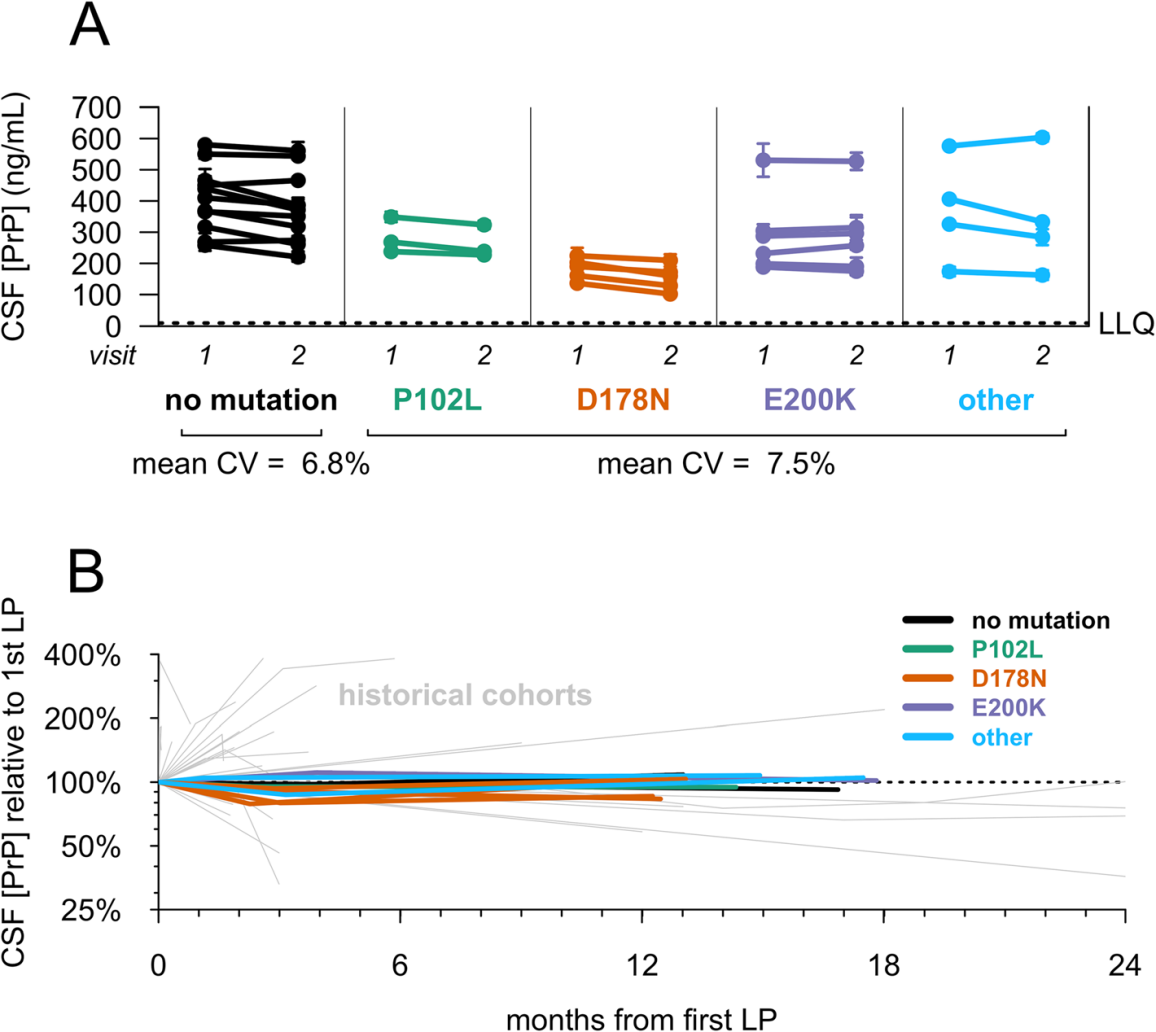
Test-retest stability of CSF PrP. Uniformly processed CSF samples were collected from lumbar punctures performed by one of two investigators (SEA, AJM). CSF PrP levels were quantified by ELISA. Dots represent means, and line segments 95% confidence intervals, of measurements within dynamic range with 2 technical replicates each. **a** Twenty-nine individuals gave two CSF samples at an interval of 2–4 months. **b** Ten participants with the noted genotypes gave three CSF samples at the following intervals: initial visit, 2–4-month follow-up visit, and 10–20-month follow-up visit. For each subject, PrP levels for all visits have been normalized to levels at the first visit, such that the first LP is defined as 100%. Gray lines show PrP test-retest stability for CSF samples from previously reported retrospective cohorts without uniform sample handling to minimize pre-analytical variability, reproduced from Vallabh et al. [25]

CSF PrP levels were stable over a 2–4-month interval and similar between carriers (mean CV = 6.8%) and controls (mean CV = 7.5%) (Fig. 1a). CSF PrP concentration differed significantly between genotypes (*p* = 0.016, one-way ANOVA), driven by lower PrP in D178N mutation carriers (Fig. 1a and Additional file 1: Table S2; see the “Discussion” section). While all reasonable efforts were made to standardize CSF collection, in some cases, clinical variations were noted, including the use of drip collection rather than aspiration and lower sample yields. On average, the six individuals whose CSF was handled differently between the two visits showed greater, though still reasonable, variation in CSF PrP levels (mean CV = 12.6%) compared to all other participants (mean CV = 5.8%). In ten individuals who completed a longitudinal study visit after 10–20 months, CSF PrP levels were again steady (mean CV of 7.2%; Fig. 1b), with variability far lower than that observed in test-retest CSF samples from retrospective cohorts lacking uniform sample handling [25] (Fig. 1b), consistent with pre-analytical factors being a major source of variability in those cohorts. CSF PrP was modestly correlated with CSF total protein (*r* = 0.35, *p* = 0.0052, two-sided Spearman’s correlation), replicating previous reports [25, 47].

**Fig. 2:**
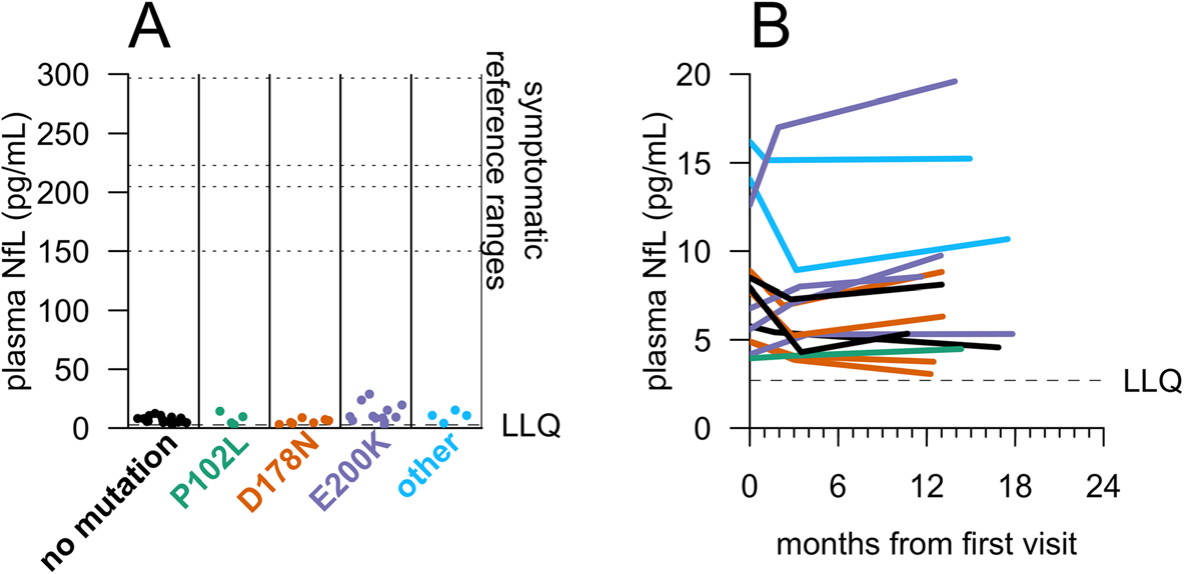
NfL levels in carrier and control plasma. Plasma NfL levels were measured by Quanterix Simoa assay. **a** For *N* = 43 participants who have made at least one study visit, samples were taken from the most recent visit at time of analysis. Dots represent singlicate measurements. Dashed lines for “symptomatic reference ranges” represent mean values reported for symptomatic genetic prion disease patients [17, 20] or median values reported for symptomatic sporadic prion disease patients [22, 23]. **b** For *N* = 14 participants who had completed three visits, plasma NfL levels were measured by Quanterix Simoa for all three visits to assess longitudinal dynamics. As in Fig. 1b, CSF from the following three timepoints is represented for each participant: initial visit, 2–4-month follow-up visit, and 10–20-month follow-up visit

In plasma, T-tau showed high variability (mean CV = 38%) over 2–4 months, limiting interpretability (Additional file 1: Figure S3A), but NfL was more reliable (mean CV = 18%; Additional file 1: Figure S3B). Groupwise, plasma NfL levels were not significantly different between controls and carriers (*p* = 0.46, two-sided Kolmogorov-Smirnov test; Fig. 2a), and all individuals were within normal ranges, well below the typical values reported in symptomatic genetic prion disease patients [17, 20]. We observed no temporal trend in plasma NfL among participants who made three visits over 10–20 months (*p* = 0.91, linear regression, Fig. 2b).

**Fig. 3:**
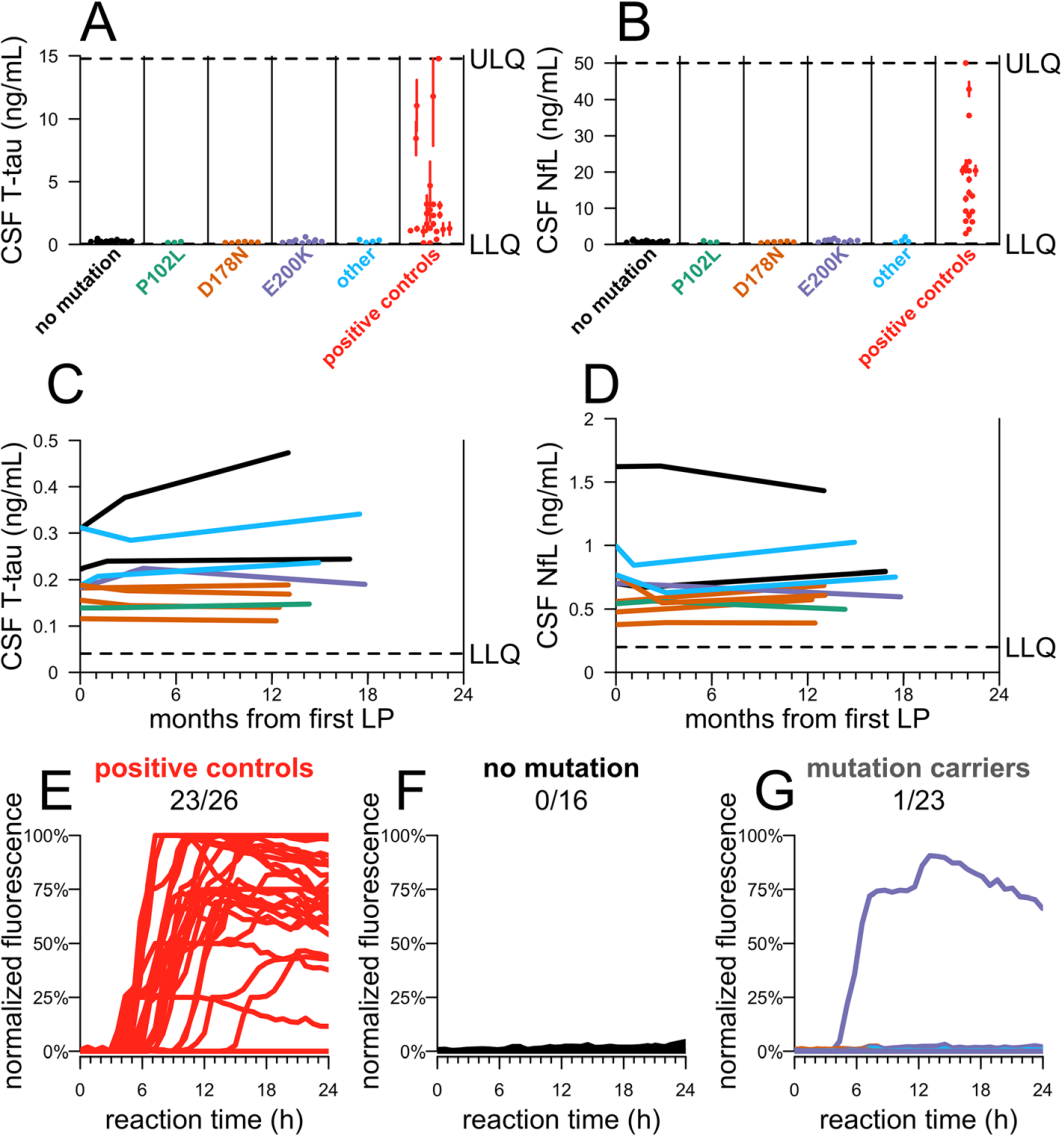
Candidate markers of neuropathology in carrier and control CSF. CSF **a** T-tau and **b** NfL levels were measured by ELISA for *N* = 39 participants who have made at least one study visit, for whom genotypes were available at time of analysis, and where appropriate CSF aliquots were available. For each participant included, samples were taken from the most recent visit at time of analysis. Symptomatic prion disease CSF samples (red, *N* = 24 for T-tau, *N* = 19 for NfL) were included from both sporadic and E200K genetic prion disease. The operator was masked to mutation status. Dots represent means, and line segments 95% confidence intervals, of measurements within dynamic range with 2 technical replicates each. **c**, **d** For *N* = 10 participants who had completed a longitudinal visit ≥ 10 months after their first visit, both T-tau and NfL were measured by ELISA across all visits to assess longitudinal dynamics. As in Fig. 1b, CSF from the following three timepoints is represented for each participant: initial visit, 2–4-month follow-up visit (the 2–4-month follow-up visit was missing for *N* = 1 participant in **c** and **d**), and 10–20-month follow-up visit. **e**–**g** RT-QuIC was performed on CSF from 39 participants who made at least one study visit. For each participant, samples were taken from the most recent visit at time of analysis. RT-QuIC was performed following an established protocol for second-generation CSF RT-QuIC using SHaPrP substrate [24]. Reactions were seeded with 20 μL CSF from *N* = 26 symptomatic prion disease cases (**e**) and *N* = 39 MGH study participants, including 16 mutation-negative (**f**) and 23 asymptomatic mutation-positive (**g**) with each reaction run in quadruplicate. Kinetic curves—normalized thioflavin T (ThT) fluorescence (*y* axis) vs. time in hours (*x* axis)—are shown for each sample, averaged across four replicates

In CSF, T-tau and NfL were highly elevated in symptomatic prion disease-positive controls (*p* = 2.6 × 10^−10^ for T-tau; *p* = 1.6 × 10^−11^ for NfL, 2-sided Kolmogorov-Smirnov test; Fig. 3a, b). In contrast, levels of these markers in mutation carrier and non-carrier control CSF were similar. CSF T-tau appeared nominally higher in non-carrier controls (mean 251.8 ± 84.5 pg/mL) than in mutation carriers (224.5 ± 112.3 pg/mL) (uncorrected *p* = 0.015, 2-sided Kolmogorov-Smirnov test), while CSF NfL was indistinguishable between the two groups (*p* = 0.61, 2-sided Kolmogorov-Smirnov test). CSF T-tau and NfL were independently measured by ELISA at a second site, and values showed good correlation with those values initially obtained (T-tau, *r* = 0.80, *p* = 3.5 × 10^−9^; NfL, *r* = 0.89. *p* = 3.0 × 10^−14^), with no difference between carriers and controls (Additional file 1: Figure S4). In *N* = 10 longitudinal participants, across all visits (10–20 months), levels of both proteins remained low with no significant change over time within individuals (*p* = 0.51 for T-tau, *p* = 0.91 for NfL, linear regression; Fig. 3c, d). The mean CV over all visits was 7.8% for CSF T-tau and 9.9% for CSF NfL.

CSF prion “seeding activity” was evaluated by RT-QuIC using two sets of previously reported conditions with pre-specified criteria [24, 46] (Fig. 3e–g and Additional file 1: Figure S5). We explored testing samples using bank vole PrP, a reportedly universal substrate for prion detection in brain tissue [46], by minimally adapting established bank vole RT-QuIC assay conditions to accommodate a larger-volume CSF input (see the “Methods” section). Under these conditions, bank vole substrate showed only 53% sensitivity (10/19 positive controls) in CSF (Additional file 1: Figure S5). However, standard diagnostic conditions [24] using Syrian hamster PrP yielded 88% sensitivity in positive controls, comparable to reported results [24] (Fig. 3e). Non-carrier control samples were negative (Fig. 3f), as were 22/23 carrier samples (Fig. 3g).

One participant, a carrier of the E200K mutation of age ≥ 60, showed RT-QuIC seeding activity upon analysis of CSF from his/her second visit (Fig. 3g), prompting comparison of all assays between both study visits (Table 2). Four of 4 RT-QuIC replicates were positive at both visits. CSF NfL remained in the normal range, comparing both to within-study normal controls and published reference ranges [17, 20]. CSF T-tau and plasma NfL were modestly higher, for both visits, than controls and other mutation carriers in our study, consistent with age (see the “Discussion” section). This participant’s score on the MRC prion disease rating scale remained stable. While their score on the Montreal Cognitive Assessment (MoCA) declined nominally between visits 1 and 2, from 27 to 25 out of a possible 30, fluctuation of one to three points was common between first and second visits in our participants, with differences of up to six points noted; furthermore, ten other participants scored 25 or less on the MoCA at least once over their first two visits. A full battery of 20 tests spanning cognitive, psychiatric, motor, and daily living assessments revealed no striking or consistent changes (Additional file 1: Table S3). This individual remained asymptomatic >1 year after his/her second visit. CSF PrP levels for this individual were in the middle of the observed range and were stable between visits 1 and 2.

**Table 2:** Comparison of visits for one RT-QuIC-positive study participant. RT-QuIC replicates were designated as positive based on the criteria described above, in the “Methods” section, and elsewhere [24]. CSF T-tau, NfL, and PrP were measured by ELISA as described in Figs. 1 and 2. Plasma NfL was measured by Simoa as described in Fig. 3

		Visit 1	Visit 2
**Fluid biomarkers**	CSF PrP (ng/mL)	287	296
	CSF T-tau (ng/mL)	0.57	0.60
	CSF NfL (ng/mL)	1.27	1.48
	Plasma NfL (pg/mL)	23.5	28.8
	RT-QuIC (positive replicates)	4/4	4/4
**Assessments**	MRC prion disease rating scale	20	20
	Montreal Cognitive Assessment	27	25

## Discussion

Here, we describe interim results from an ongoing longitudinal cohort study characterizing genetic prion disease mutation carriers and mutation-negative controls. We evaluate candidate fluid biomarkers for primary and secondary prevention trial designs in pre-symptomatic genetic prion disease.

PrP-lowering therapeutics are now in preclinical development, so CSF PrP will be important as a pharmacodynamic biomarker at a minimum, and regulators have expressed openness to its use as a surrogate endpoint in pre-symptomatic individuals [10]. Productive use of this marker in trials, however, will require dependable performance, including a stable baseline, which might not exist if CSF PrP exhibits high biotemporal variability or if pre-symptomatic individuals exhibit a decline in CSF PrP, similar to the lowered CSF PrP levels seen in symptomatic prion disease patients [25, 47, 48]. Our findings address both of these concerns.

Most historical cohorts in which CSF PrP has been evaluated exhibit large variability in CSF PrP levels [21, 25, 48], but we observed tight 8–11-week test-retest reliability (mean CV = 13%) among uniformly handled samples, suggesting that PrP can be reliably measured if pre-analytical variables are minimized. Here, we validate this hypothesis prospectively, observing a mean CV of only 7% over 10–20 months among samples handled with exquisite uniformity and early addition of detergent to minimize adsorption to plasticware. This biotemporal stability is comparable to that of core CSF biomarkers including amyloid beta (Aβ) 1–38, Aβ 1–40, T-tau, and NfL over a similar term [49]. In addition, whereas our previous report was based on patients suffering from non-prion mild cognitive impairment [25], in whom progressive reduction of CSF PrP due to disease would not be expected, here, we confirm that CSF PrP concentration is stable even among individuals at high risk of developing prion disease in their lifetimes.

PrP levels in the CSF of symptomatic D178N individuals have been reported to be lower than those of individuals with other *PRNP* mutations or no mutation [48], a finding replicated here among pre-symptomatic carriers. This difference has been interpreted by some to indicate a prodromal disease process underway [48]; however, several reports show preferential degradation of D178N mutant PrP, independent of the disease process [50–53]. Here, lower CSF PrP levels in D178N carriers appear to be stable over short (Fig. 1a) and longer terms (Fig. 1b), suggesting that this reduced level is constitutive rather than a prodromal change. Stability of CSF PrP levels even in one E200K mutation carrier with RT-QuIC seeding activity (Table 2) suggests that the decline in CSF PrP levels seen in symptomatic disease likely emerges later in the disease process and should not confound CSF PrP stability in asymptomatic carriers without observable pathology.

Broadly, these findings suggest that CSF PrP levels are stable enough in any one individual, regardless of *PRNP* mutation, to informatively report on a PrP-lowering therapeutic such as a PrP-lowering antisense oligonucleotide (ASO) in the central nervous system, over time frames likely to be of relevance to dose-finding and biomarker-based trials. In a phase I/II trial of the huntingtin-lowering ASO tominersen, mutant huntingtin protein was reduced by a mean of 40% in CSF in the two highest dose cohorts [54]; our data suggest that a similar reduction in PrP levels would be reliably detectable in CSF. The LP tolerability data we were able to collect suggests that intrathecal delivery of a drug will not be a barrier to treatment among pre-symptomatic carriers of *PRNP* mutations. While our study is biased toward highly motivated carriers willing to participate in research, this same bias will likely apply to trial recruitment, supporting the relevance of these data.

Across the candidate disease markers measured, most carriers could not be distinguished from non-carrier controls. Put differently, the present data do not support analogies between the disease state of most adult carriers and the clinically silent incubation phase in prion-infected animals [55–59]. Previous cohort studies and case reports have largely found imaging and physiological changes to coincide with onset [14–16, 60–63]; reports of suggestive MRI and biochemical changes in single individuals have been confined to the 1 to 2 years before symptom onset [14, 17–19]. Our findings are in keeping with this trend of limited changes before symptom onset: prion seeding activity was detected in just 1/23 pre-symptomatic carriers, with neuronal damage markers just slightly higher than other participants, but still within the range of aged healthy controls [64–66] and well below typical values for symptomatic prion disease [17, 20, 22, 23, 67]. Given that this individual remained asymptomatic at the last follow-up, the prognostic value of a positive RT-QuIC result in an asymptomatic individual remains unclear.

To our knowledge, ours is the first report of RT-QuIC seeding activity in the absence of prion disease symptom onset. These data may suggest that for individuals harboring the E200K mutation, RT-QuIC seeding activity may offer an early sign of pathological change, before behavioral or cognitive scales can detect changes in performance. But this finding should be interpreted with caution. One previous report found that an E200K individual converted from RT-QuIC negative to positive between 2 and 4 months after symptom onset [68]. A report of a sporadic CJD patient who happened to undergo CSF RT-QuIC before and after onset found the test to be positive only after onset [19]. These differences could reflect differences in RT-QuIC protocols employed, or variability in conversion time relative to symptom identification between individuals. Overall, in our hands, 9/10 E200K carriers and 13/13 carriers of other mutations were negative by RT-QuIC, suggesting that detectable seeding activity is unlikely to be consistently present for a long period prior to onset.

Our study has several limitations. As discussed above, orthogonal lines of evidence lend support to the biological validity of our observation that D178N mutation carriers have lower groupwise CSF PrP levels compared to other participants in our study. However, based on our data alone, we cannot exclude the possibility that the ELISA we used to measure CSF PrP may be differentially reactive to some mutant forms of the protein. Our study relied on positive control samples from symptomatic CJD patients collected at various clinical centers. As we have previously shown that heterogenous CSF sample handling precludes reliable quantification of CSF PrP [25], we were therefore unable to include symptomatic samples in our CSF PrP analysis. In our assessment of disease biomarkers, we focused on a small set of markers with the best-established association with the prion disease process. Future analyses could explore other emerging biomarkers of neurodegeneration, such as synaptic proteins [69, 70].

Our study is relatively young, and our analyses to date provide only short- to medium-term and cross-sectional findings. Moving forward, participants will be seen at annual intervals where feasible, with an eye to enhancing the longitudinal analysis of CSF PrP and enabling longitudinal tracking of pathological biomarkers. Complementary perspectives on the above may be provided by two other efforts, as yet unpublished, to systematically characterize healthy individuals carrying genetic prion disease predisposing mutations: the UK National Prion Monitoring Cohort and UCSF Early Diagnosis of Human Prion Disease studies [38, 71]. Age of onset is highly variable in genetic prion disease, we have no means to predict time to onset for any individual carrier in our cohort, and annual hazard for any given individual is low [9]; prior experience suggests that observing even a handful of conversions in a prospective carrier cohort could take between 10 and 20 years. For this reason, in the near term, a study of this nature is better positioned to report on the state of the average carrier at a given time than on the dynamics of conversion to the disease state.

A strength of our study is that the highly penetrant prion disease-causing mutations most common in the general population [7] are also those most represented in our cohort. Our characterization may therefore provide a reasonable cross-sectional snapshot of carriers available for recruitment for research or trials.

## Conclusion

The above characterization of a cohort of pre-symptomatic genetic prion disease mutation carriers and controls suggests that CSF PrP levels are stable in the carrier population and may therefore serve as a meaningful biomarker for PrP-lowering therapeutics. In the meantime, at present, our findings regarding disease stage biomarkers suggest that a “secondary prevention” strategy may not be feasible in genetic prion disease: any prodromal period may be too subtle, too brief, or present in too few individuals at any given time to enable recruitment of a large enough prodromal cohort to enable trials. It remains possible that a fluid biomarker that reliably presages symptom onset further in advance could emerge from further study, particularly in more slowly progressive genetic prion disease variants, allowing subclinical pathology to be tracked in a small cohort of carriers and leveraged toward a secondary prevention trial design. However, our present findings may reflect where the field is likely to stand as therapeutics presently in development approach clinical trials. In this context, pre-symptomatic trials in genetic prion disease may be better served by a primary prevention model based on genetic risk.

## Supplementary information

**Additional file 1: Figure S1.** Overview of study participant inclusion and exclusion. **Figure S2.** Pre- and post-procedure anxiety in participants experiencing their first lumbar puncture. **Figure S3.** Short-term test-retest stability of markers of neuronal damage in carrier and control plasma. **Figure S4.** Additional measurements and statistics on markers of neuronal damage in carrier and control CSF. **Figure S5.** RT-QuIC results with recombinant bank vole PrP. **Table S1.** Measures of cognitive, psychiatric, motor and daily functioning in all MGH study participants. **Table S2.** CSF PrP levels by mutation status. **Table S3.** Additional measures of cognitive, psychiatric, motor and daily functioning for one RT-QuIC positive MGH study participant.

## Data Availability

To protect participant confidentiality, study data cannot be made publicly available, but de-identified data will be provided to qualified investigators upon request. R source code and study documents are available in a public repository: https://github.com/ericminikel/mgh_prnp_fluid_biomarkers.

## Abbreviations

ASO: Antisense oligonucleotide
Aβ: Amyloid beta
CSF: Cerebrospinal fluid
CV: Coefficient of variation
ELISA: Enzyme-linked immunosorbent assay
NfL: Neurofilament light
*PRNP*: The prion protein gene
PrP: Prion protein
RT-QuIC: Real-time quaking-induced conversion
T-tau: Total tau

## References

[1] Prusiner SB (1998). Prions. PNAS.

[2] Raymond GJ, Zhao HT, Race B, Raymond LD, Williams K, Swayze EE, et al. Antisense oligonucleotides extend survival of prion-infected mice. JCI Insight. 2019;5. 10.1172/jci.insight.131175. 10.1172/jci.insight.131175 PMC6777807 31361599

[3] Minikel EV, Zhao HT, Le J, O’Moore J, Pitstick R, Graffam S, et al. Prion protein lowering is a disease-modifying therapy across prion strains, disease stages, and endpoints. bioRxiv. 2020. 10.1101/2020.03.27.011940. 10.1093/nar/gkaa616 PMC7641729 32776089

[4] Pocchiari M, Puopolo M, Croes EA, Budka H, Gelpi E, Collins S (2004). Predictors of survival in sporadic Creutzfeldt-Jakob disease and other human transmissible spongiform encephalopathies. Brain.

[5] Geschwind MD, Kuo AL, Wong KS, Haman A, Devereux G, Raudabaugh BJ (2013). Quinacrine treatment trial for sporadic Creutzfeldt-Jakob disease. Neurology.

[6] Haïk S, Marcon G, Mallet A, Tettamanti M, Welaratne A, Giaccone G (2014). Doxycycline in Creutzfeldt-Jakob disease: a phase 2, randomised, double-blind, placebo-controlled trial. Lancet Neurol.

[7] Minikel EV, Vallabh SM, Lek M, Estrada K, Samocha KE, Sathirapongsasuti JF (2016). Quantifying prion disease penetrance using large population control cohorts. Sci Transl Med.

[8] Owen J, Beck J, Campbell T, Adamson G, Gorham M, Thompson A (2014). Predictive testing for inherited prion disease: report of 22 years experience. Eur J Hum Genet.

[9] Minikel EV, Vallabh SM, Orseth MC, Brandel J-P, Haïk S, Laplanche J-L, et al. Age at onset in genetic prion disease and the design of preventive clinical trials. Neurology. 2019;93(2):e125–e134. 10.1212/WNL.0000000000007745. 10.1212/WNL.0000000000007745 PMC6656649 31171647

[10] Vallabh SM, Minikel EV, Schreiber SL, Lander ES (2020). Towards a treatment for genetic prion disease: trials and biomarkers. Lancet Neurol.

[11] Sperling R, Mormino E, Johnson K (2014). The evolution of preclinical Alzheimer’s disease: implications for prevention trials. Neuron.

[12] Bateman RJ, Xiong C, Benzinger TLS, Fagan AM, Goate A, Fox NC (2012). Clinical and biomarker changes in dominantly inherited Alzheimer’s disease. N Engl J Med.

[13] Ross CA, Aylward EH, Wild EJ, Langbehn DR, Long JD, Warner JH (2014). Huntington disease: natural history, biomarkers and prospects for therapeutics. Nat Rev Neurol.

[14] Cortelli P, Perani D, Montagna P, Gallassi R, Tinuper P, Provini F (2006). Pre-symptomatic diagnosis in fatal familial insomnia: serial neurophysiological and 18FDG-PET studies. Brain.

[15] Cohen OS, Chapman J, Korczyn AD, Nitsan Z, Appel S, Hoffmann C (2015). Familial Creutzfeldt-Jakob disease with the E200K mutation: longitudinal neuroimaging from asymptomatic to symptomatic CJD. J Neurol.

[16] Rudge P, Jaunmuktane Z, Hyare H, Ellis M, Koltzenburg M, Collinge J, et al. Early neurophysiological biomarkers and spinal cord pathology in inherited prion disease. Brain. 2019;142(3):760–70. 10.1093/brain/awy358. 10.1093/brain/awy358 PMC6391599 30698738

[17] Steinacker P, Blennow K, Halbgebauer S, Shi S, Ruf V, Oeckl P (2016). Neurofilaments in blood and CSF for diagnosis and prediction of onset in Creutzfeldt-Jakob disease. Sci Rep.

[18] Abu-Rumeileh S, Steinacker P, Polischi B, Mammana A, Bartoletti-Stella A, Oeckl P (2019). CSF biomarkers of neuroinflammation in distinct forms and subtypes of neurodegenerative dementia. Alzheimers Res Ther.

[19] Novi G, Canosa A, Nobili F, Bongianni M, Zanusso G, Balestrino M (2018). Longitudinal brain magnetic resonance imaging and real-time quaking induced conversion analysis in presymptomatic Creutzfeldt-Jakob disease. Eur J Neurol.

[20] Kovacs GG, Andreasson U, Liman V, Regelsberger G, Lutz MI, Danics K (2017). Plasma and cerebrospinal fluid tau and neurofilament concentrations in rapidly progressive neurological syndromes: a neuropathology-based cohort. Eur J Neurol.

[21] Abu Rumeileh S, Lattanzio F, Stanzani Maserati M, Rizzi R, Capellari S, Parchi P. Diagnostic accuracy of a combined analysis of cerebrospinal fluid t-PrP, t-tau, p-tau, and Aβ42 in the differential diagnosis of Creutzfeldt-Jakob disease from Alzheimer’s disease with emphasis on atypical disease variants. J Alzheimers Dis. 2017;55:1471–80. 10.3233/JAD-160740 PMC5181677 27886009

[22] Thompson AGB, Luk C, Heslegrave AJ, Zetterberg H, Mead SH, Collinge J, et al. Neurofilament light chain and tau concentrations are markedly increased in the serum of patients with sporadic Creutzfeldt-Jakob disease, and tau correlates with rate of disease progression. J Neurol Neurosurg Psychiatry. 2018;89(9):955–61. 10.1136/jnnp-2017-317793. 10.1136/jnnp-2017-317793 PMC6109239 29487167

[23] Staffaroni AM, Kramer AO, Casey M, Kang H, Rojas JC, Orrú CD (2019). Association of blood and cerebrospinal fluid tau level and other biomarkers with survival time in sporadic Creutzfeldt-Jakob disease. JAMA Neurol.

[24] Orrú CD, Groveman BR, Hughson AG, Zanusso G, Coulthart MB, Caughey B. Rapid and sensitive RT-QuIC detection of human Creutzfeldt-Jakob disease using cerebrospinal fluid. MBio. 2015;6(1). 10.1128/mBio.02451-14. 10.1128/mBio.02451-14 PMC4313917 25604790

[25] Vallabh SM, Nobuhara CK, Llorens F, Zerr I, Parchi P, Capellari S (2019). Prion protein quantification in human cerebrospinal fluid as a tool for prion disease drug development. PNAS.

[26] Harris PA, Taylor R, Minor BL, Elliott V, Fernandez M, O’Neal L (2019). The REDCap consortium: building an international community of software platform partners. J Biomed Inform.

[27] Nasreddine ZS, Phillips NA, Bédirian V, Charbonneau S, Whitehead V, Collin I (2005). The Montreal Cognitive Assessment, MoCA: a brief screening tool for mild cognitive impairment. J Am Geriatr Soc.

[28] Homack S, Lee D, Riccio CA. Test review: Delis-Kaplan executive function system. J Clin Exp Neuropsychol. 2005;27(5):599–609. 10.1080/13803390490918444 16019636

[29] Strauss E, Sherman EMS, Spreen O. A compendium of neuropsychological tests: administration, norms and commentary. 3rd ed. Oxford: Oxford University Press; 2006.

[30] Reitan RM, Wolfson D (1993). Halstead-Reitan neuropsychological battery.

[31] Souillard-Mandar W, Davis R, Rudin C, Au R, Libon DJ, Swenson R (2016). Learning classification models of cognitive conditions from subtle behaviors in the digital clock drawing test. Mach Learn.

[32] Weintraub S, Dikmen SS, Heaton RK, Tulsky DS, Zelazo PD, Bauer PJ (2013). Cognition assessment using the NIH Toolbox. Neurology.

[33] Leyfer OT, Ruberg JL, Woodruff-Borden J (2006). Examination of the utility of the Beck Anxiety Inventory and its factors as a screener for anxiety disorders. J Anxiety Disord.

[34] Beck AT, Steer RA, Ball R, Ranieri WF (1996). Comparison of Beck depression inventories-IA and-II in psychiatric outpatients. J Pers Assess.

[35] Farias ST, Mungas D, Reed BR, Cahn-Weiner D, Jagust W, Baynes K (2008). The Measurement of Everyday Cognition (ECog): scale development and psychometric properties. Neuropsychology.

[36] Farias ST, Mungas D, Harvey DJ, Simmons A, Reed BR, DeCarli C (2011). The Measurement of Everyday Cognition (ECog): development and validation of a short form. Alzheimers Dement.

[37] Johns MW (1991). A new method for measuring daytime sleepiness: the Epworth sleepiness scale. Sleep.

[38] Thompson AGB, Lowe J, Fox Z, Lukic A, Porter M-C, Ford L (2013). The Medical Research Council prion disease rating scale: a new outcome measure for prion disease therapeutic trials developed and validated using systematic observational studies. Brain.

[39] Goetz CG, Tilley BC, Shaftman SR, Stebbins GT, Fahn S, Martinez-Martin P, et al. Movement Disorder Society-sponsored revision of the unified Parkinson’s disease rating scale (MDS-UPDRS): scale presentation and clinimetric testing results. Mov Disord. 2008;23:2129–70. 10.1002/mds.22340 19025984

[40] Cummings JL, Mega M, Gray K, Rosenberg-Thompson S, Carusi DA, Gornbein J (1994). The Neuropsychiatric Inventory: comprehensive assessment of psychopathology in dementia. Neurology.

[41] Capellari S, Vital C, Parchi P, Petersen RB, Ferrer X, Jarnier D (1997). Familial prion disease with a novel 144-bp insertion in the prion protein gene in a Basque family. Neurology.

[42] Dorey A, Tholance Y, Vighetto A (2015). Association of cerebrospinal fluid prion protein levels and the distinction between Alzheimer disease and Creutzfeldt-Jakob disease. JAMA Neurol.

[43] Gaetani L, Höglund K, Parnetti L, Pujol-Calderon F, Becker B, Eusebi P, et al. A new enzyme-linked immunosorbent assay for neurofilament light in cerebrospinal fluid: analytical validation and clinical evaluation. Alzheimers Res Ther. 2018;10. 10.1186/s13195-018-0339-1. 10.1186/s13195-018-0339-1 PMC6389166 29370869

[44] Gisslén M, Price RW, Andreasson U, Norgren N, Nilsson S, Hagberg L (2015). Plasma concentration of the neurofilament light protein (NFL) is a biomarker of CNS injury in HIV infection: a cross-sectional study. EBioMedicine.

[45] Orrù CD, Groveman BR, Hughson AG, Manca M, Raymond LD, Raymond GJ (1658). RT-QuIC assays for prion disease detection and diagnostics. Methods Mol Biol.

[46] Orrú CD, Groveman BR, Raymond LD, Hughson AG, Nonno R, Zou W, et al. Bank vole prion protein as an apparently universal substrate for RT-QuIC-based detection and discrimination of prion strains. PLoS Pathog. 2015;11(6):e1004983. 10.1371/journal.ppat.1004983. 10.1371/journal.ppat.1004983 PMC4472236 26086786

[47] Minikel EV, Kuhn E, Cocco AR, Vallabh SM, Hartigan CR, Reidenbach AG, et al. Domain-specific quantification of prion protein in cerebrospinal fluid by targeted mass spectrometry. Mol Cell Proteomics. 2019;18(12):2388–400. 10.1074/mcp.RA119.001702. 10.1074/mcp.RA119.001702 PMC6885701 31558565

[48] Villar-Piqué A, Schmitz M, Lachmann I, Karch A, Calero O, Stehmann C, et al. Cerebrospinal fluid total prion protein in the spectrum of prion diseases. Mol Neurobiol. 2018. 10.1007/s12035-018-1251-1 30062673

[49] Trombetta BA, Carlyle BC, Koenig AM, Shaw LM, Trojanowski JQ, Wolk DA, et al. The technical reliability and biotemporal stability of cerebrospinal fluid biomarkers for profiling multiple pathophysiologies in Alzheimer’s disease. PLoS One. 2018;13. 10.1371/journal.pone.0193707. 10.1371/journal.pone.0193707 PMC5837100 29505610

[50] Parchi P, Petersen RB, Chen SG, Autilio-Gambetti L, Capellari S, Monari L (1998). Molecular pathology of fatal familial insomnia. Brain Pathol.

[51] Petersen RB, Parchi P, Richardson SL, Urig CB, Gambetti P (1996). Effect of the D178N mutation and the codon 129 polymorphism on the metabolism of the prion protein. J Biol Chem.

[52] Jackson WS, Borkowski AW, Faas H, Steele AD, King OD, Watson N (2009). Spontaneous generation of prion infectivity in fatal familial insomnia knockin mice. Neuron.

[53] Watts JC, Giles K, Bourkas MEC, Patel S, Oehler A, Gavidia M (2016). Towards authentic transgenic mouse models of heritable PrP prion diseases. Acta Neuropathol.

[54] Tabrizi SJ, Leavitt BR, Landwehrmeyer GB, Wild EJ, Saft C, Barker RA, et al. Targeting huntingtin expression in patients with Huntington’s disease. N Engl J Med. 2019. 10.1056/NEJMoa1900907. 10.1056/NEJMoa1900907 31059641

[55] Orrù CD, Hughson AG, Race B, Raymond GJ, Caughey B (2012). Time course of prion seeding activity in cerebrospinal fluid of scrapie-infected hamsters after intratongue and intracerebral inoculations. J Clin Microbiol.

[56] Tamgüney G, Francis KP, Giles K, Lemus A, DeArmond SJ, Prusiner SB (2009). Measuring prions by bioluminescence imaging. Proc Natl Acad Sci U S A.

[57] Hwang D, Lee IY, Yoo H, Gehlenborg N, Cho J-H, Petritis B (2009). A systems approach to prion disease. Mol Syst Biol.

[58] Hirouchi M (2019). Neurofilament light chain (NfL) as a possible biomarker for drug efficacy in mouse models of neurodegenerative diseases.

[59] Llorens F, Barrio T, Correia Â, Villar-Piqué A, Thüne K, Lange P (2018). Cerebrospinal fluid prion disease biomarkers in pre-clinical and clinical naturally occurring scrapie. Mol Neurobiol.

[60] Satoh K, Nakaoke R, Nishiura Y, Tsujino A, Motomura M, Yoshimura T (2011). Early detection of sporadic CJD by diffusion-weighted MRI before the onset of symptoms. J Neurol Neurosurg Psychiatry.

[61] Verde F, Ticozzi N, Messina S, Calcagno N, Girotti F, Maderna L (2016). MRI abnormalities found 1 year prior to symptom onset in a case of Creutzfeldt-Jakob disease. J Neurol.

[62] Terasawa Y, Fujita K, Izumi Y, Kaji R (2012). Early detection of familial Creutzfeldt-Jakob disease on diffusion-weighted imaging before symptom onset. J Neurol Sci.

[63] Zanusso G, Camporese G, Ferrari S, Santelli L, Bongianni M, Fiorini M (2016). Long-term preclinical magnetic resonance imaging alterations in sporadic Creutzfeldt-Jakob disease. Ann Neurol.

[64] Mattsson N, Rosén E, Hansson O, Andreasen N, Parnetti L, Jonsson M (2012). Age and diagnostic performance of Alzheimer disease CSF biomarkers. Neurology..

[65] Mattsson N, Andreasson U, Zetterberg H, Blennow K (2017). Alzheimer’s Disease Neuroimaging Initiative. Association of plasma neurofilament light with neurodegeneration in patients with Alzheimer disease. JAMA Neurol.

[66] Sjögren M, Vanderstichele H, Agren H, Zachrisson O, Edsbagge M, Wikkelsø C (2001). Tau and Abeta42 in cerebrospinal fluid from healthy adults 21-93 years of age: establishment of reference values. Clin Chem.

[67] Abu-Rumeileh S, Capellari S, Stanzani-Maserati M, Polischi B, Martinelli P, Caroppo P (2018). The CSF neurofilament light signature in rapidly progressive neurodegenerative dementias. Alzheimers Res Ther.

[68] Sano K, Satoh K, Atarashi R, Takashima H, Iwasaki Y, Yoshida M (2013). Early detection of abnormal prion protein in genetic human prion diseases now possible using real-time QUIC assay. PLoS One.

[69] Blennow K, Diaz-Lucena D, Zetterberg H, Villar-Pique A, Karch A, Vidal E (2019). CSF neurogranin as a neuronal damage marker in CJD: a comparative study with AD. J Neurol Neurosurg Psychiatry.

[70] Colom-Cadena M, Spires-Jones T, Zetterberg H, Blennow K, Caggiano A, DeKosky ST (2020). The clinical promise of biomarkers of synapse damage or loss in Alzheimer’s disease. Alz Res Therapy.

[71] Takada LT, Kim M-O, Cleveland RW, Wong K, Forner SA, Gala II (2017). Genetic prion disease: experience of a rapidly progressive dementia center in the United States and a review of the literature. Am J Med Genet B Neuropsychiatr Genet.

