# Supplementary material for: Cerebrospinal fluid and plasma biomarkers in individuals at risk for genetic prion disease
Source: BMC Med. 2020 Jun 18;18:140. doi: 10.1186/s12916-020-01608-8 (PMC7302371; doi:10.1186/s12916-020-01608-8)
Supplement: Supplementary file 1 — Additional file 1: Figure S1. Overview of study participant inclusion and exclusion. Figure S2. Pre- and post-procedure anxiety in participants experiencing their first lumbar puncture. Figure S3. Short-term test-retest stability of markers of neuronal damage in carrier and control plasma. Figure S4. Additional measurements and statistics on markers of neuronal damage in carrier and control CSF. Figure S5. RT-QuIC results with recombinant bank vole PrP. Table S1. Measures of cognitive, psychiatric, motor and daily functioning in all MGH study participants. Table S2. CSF PrP levels by mutation status. Table S3. Additional measures of cognitive, psychiatric, motor and daily functioning for one RT-QuIC positive MGH study participant. [file 12916_2020_1608_MOESM1_ESM.docx]

**ADDITIONAL FILE 1**

**Cerebrospinal fluid and plasma biomarkers in individuals at risk for genetic prion disease**

Sonia M Vallabh^1,2,3,4,†^, Eric Vallabh Minikel^1,2,3,4^, Victoria J Williams^2^, Becky C Carlyle^2^,

Alison J McManus^2^, Chase D Wennick^2^, Anna Bolling^2^, Bianca A Trombetta^2^, David Urick^2^, Chloe K Nobuhara^2^, Jessica Gerber^2^, Holly Duddy^2^, Ingolf Lachmann^5^, Christiane Stehmann^6^, Steven J Collins^6^, Kaj Blennow^7^, Henrik Zetterberg^7,8,9,10^, Steven E Arnold^1,2,†^

Table of Contents

Supplementary Figures 2

Supplementary Tables 6

STROBE Checklist 8

# Supplementary Figures

******

***Figure S1: Overview of study participant inclusion and exclusion.*** Of N=69 volunteers, N=43 completed at least one study visit, and N=34 participated in ongoing study visits at the pre-specified intervals.

**
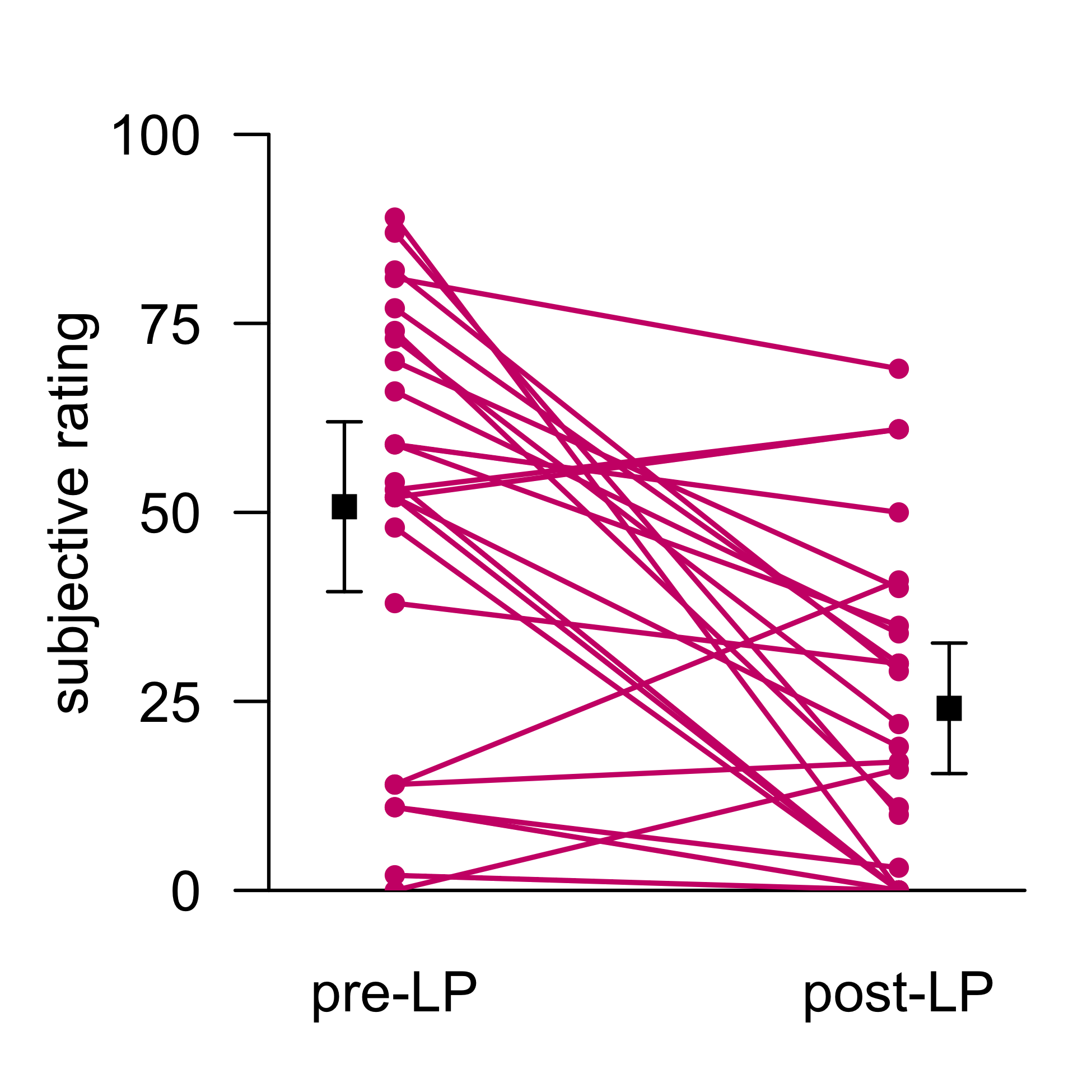
**

***Figure S2: Pre- and post-procedure anxiety in participants experiencing their first lumbar puncture.*** *Following the lumbar puncture procedure, participants were asked to quantitatively rank both their anxiety before the procedure and their post-procedure anxiety when contemplating a future LP, using a Likert-type scale. Responses are normalized to the length of the scale and shown only for first study visits, for N=24 individuals who had never undergone a previous LP.*

**
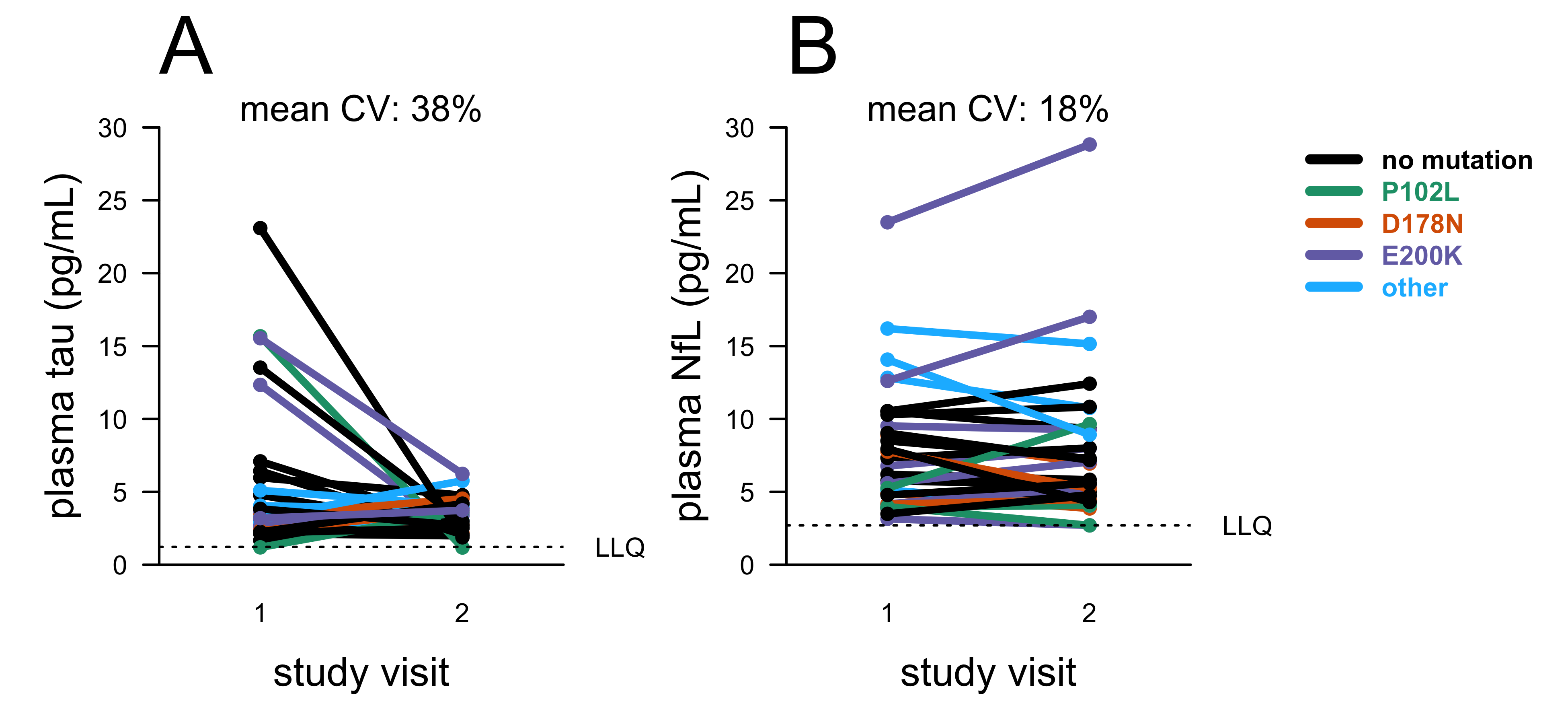
**

***Figure S3: Short-term test-retest stability of markers of neuronal damage in carrier and control plasma.*** *Plasma T-tau and NfL levels were measured by Quanterix Simoa assay at University of Gothenburg. For N=30 participants (T-tau) or N=31 participants (NfL) who have made at least two study visits, samples were taken from the initial two study visits, completed 2-4 months apart. Dots represent singlicate measurements. For participants with exactly two visits, the NfL measurement from the second visit displayed here is also displayed in Figure 2.*

**
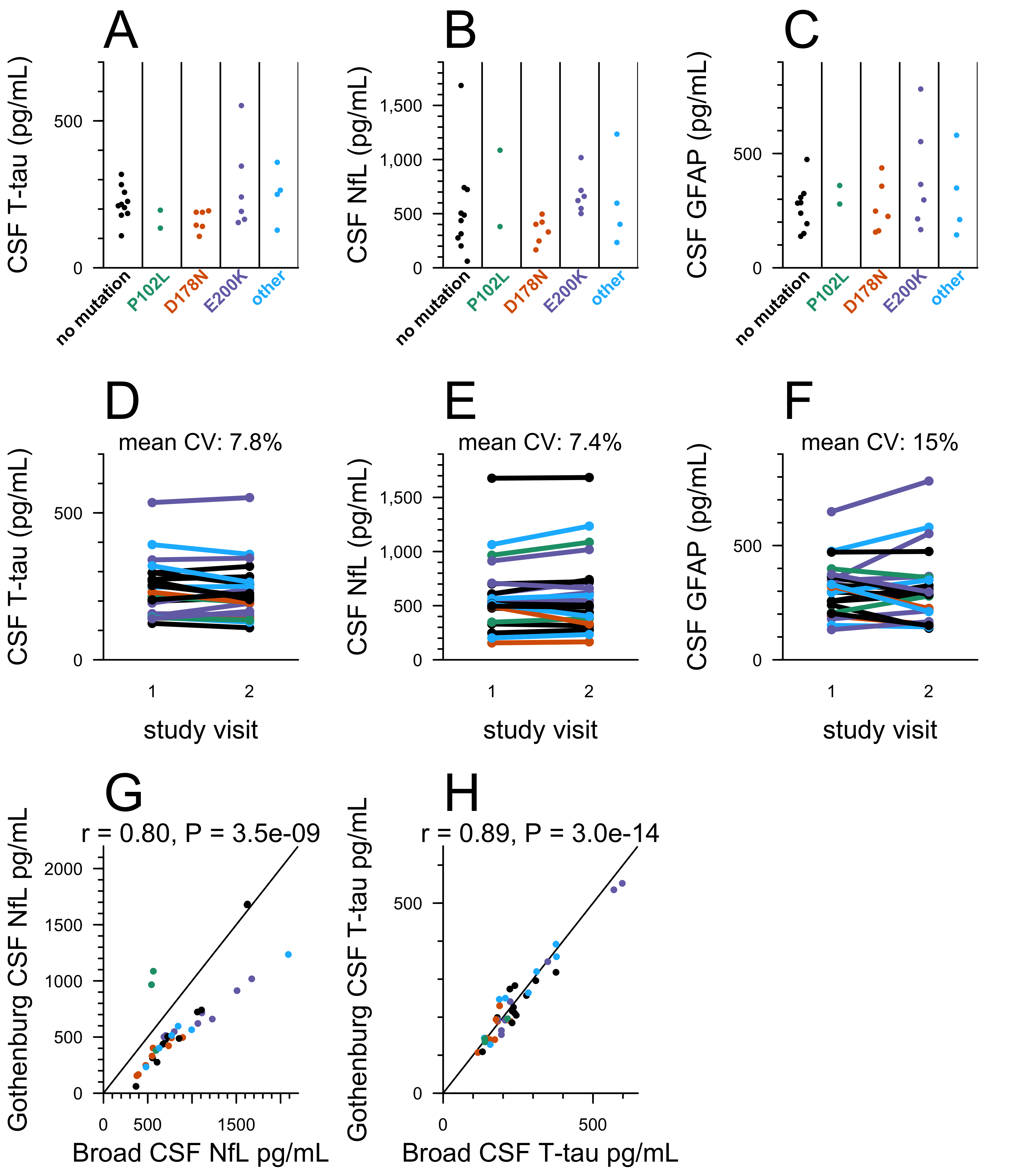
**

***Figure S4: Additional measurements and statistics on markers of neuronal damage in carrier and control CSF.*** *CSF* ***A)*** *T-tau and* ***B)*** *NfL and C) glial fibrillary acidic protein (GFAP) were measured by ELISA at University of Gothenburg for N=28 participants who had made at least one study visit, for whom genotypes were available at time of analysis, and where appropriate CSF aliquots were available. For each participant included, samples were taken from the most recent visit at time of analysis. The operator was masked to mutation status. Dots represent means, and line segments 95% confidence intervals, of measurements within dynamic range with 2 technical replicates each.* ***D-F)*** *For N=22 participants who had completed two visits at time of analysis, both total tau and NfL were measured by ELISA at University of Gothenburg for both visits to assess within-subject variability over a 2-4 month timeframe.* ***G-H****) Correlation between results obtained from two independent ELISA analyses at different sites (Broad Institute and University of Gothenburg; assays performed using different methods for NfL, and the same commercial kit for T-tau; see Methods) for N=36 samples from N=27 individuals (G) and N=39 samples from N=27 individuals (H).*


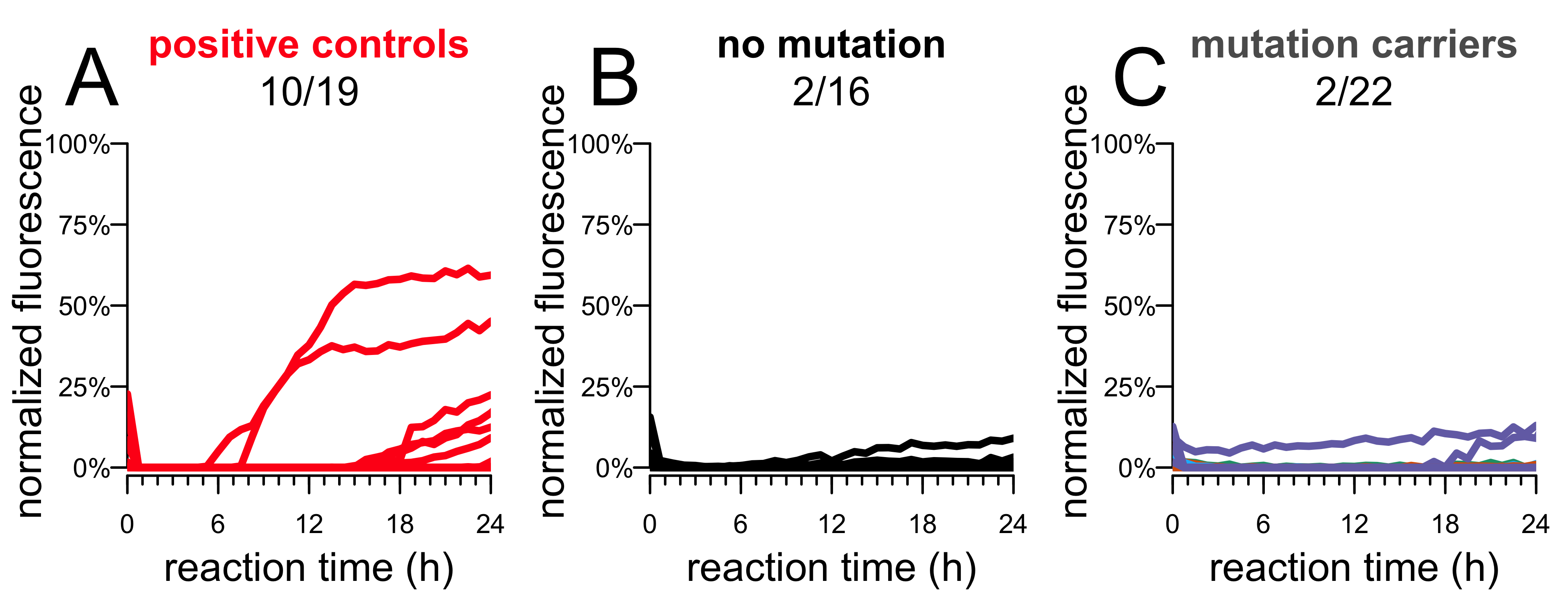


***Figure S5: RT-QuIC results with recombinant bank vole PrP.*** *RT-QuIC* *was performed on CSF from 39 participants who have made at least one study visit, selected to match the samples used in the SHaPrP RT-QuIC analysis (Figure 3E-G). RT-QuIC was performed following a published protocol for RT-QuIC using BvPrP substrate, intended for prion detection in brain homogenate. Reagent concentrations were adjusted to accommodate 20 μL CSF in a final reaction volume of 100 μL. N=22 prion disease cases or N=39 MGH study participants were assayed, with each reaction run in quadruplicate. Kinetic curves – normalized thioflavin T (ThT) fluorescence (y axis) vs. time in hours (x axis) – are shown for each replicate.*

# Supplementary Tables

***Table S1.*** ***Measures of cognitive, psychiatric, motor and daily functioning in all MGH study participants.*** *Scores are averaged across group (carrier vs. non-carrier) at first completed study visit. P values are from two-sided Kolmogorov-Smirnov tests. Bonferroni corrected p values account for a multiple testing burden of N=20 for all measures shown. Raw scores are provided according to the methods standard for each test, with the exception of the NIH Toolbox overall cognition composite score, and the digital clock drawing test, where digitally recorded features were compiled into a composite score as previously described* [31]*.*

|  | | ***PRNP* mutation carriers** | **Non-carrier controls** | **P value (raw)** | **P value (Bonferroni corrected)** |
| --- | --- | --- | --- | --- | --- |
| **Assessment scores** | Montreal Cognitive Assessment | 27.6±1.6 | 28.4±1.8 | 0.16 | 1 |
|  | NIH Toolbox Overall Cognition | 103.5±12.1 | 115.2±14.1 | 0.029 | 0.59 |
|  | Digital clock-drawing test | 73.8±20.3 | 73.5±23.7 | 0.58 | 1 |
|  | Trailmaking test, Part A | 21.7±7.0 | 22.0±8.2 | 0.31 | 1 |
|  | Trailmaking test, Part B | 57.3±29.4 | 46.6±11.8 | 0.22 | 1 |
|  | DKEFS Color-word interference test, inhibition time | 53.0±13.7 | 49.9±11.6 | 0.58 | 1 |
|  | DKEFS Color-word interference test, switching time | 60.3±14.6 | 57.6±16.4 | 0.52 | 1 |
|  | DKEFS Letter Fluency | 40.3±12.6 | 48.2±10.1 | 0.11 | 1 |
|  | DKEFS Category Fluency | 42.7±10.5 | 44.9±10.8 | 0.46 | 1 |
|  | DKEFS Category Switching | 14.6±2.5 | 15.7±2.2 | 0.9 | 1 |
|  | Grooved pegboard test, dominant hand | 72.2±15.1 | 62.9±9.6 | 0.062 | 1 |
|  | Grooved pegboard test, non-dominant hand | 82.0±21.5 | 69.1±14.5 | 0.018 | 0.36 |
|  | RAVLT – delayed recall | 8.8±3.2 | 10.0±2.8 | 0.42 | 1 |
| **Questionnaire scores** | MRC prion disease rating scale | 20.0±0.2 | 20.0±0.0 | 1 | 1 |
|  | Epworth Sleepiness Scale | 6.4±3.5 | 5.4±3.5 | 0.84 | 1 |
|  | Athens Insomnia Scale | 5.9±4.6 | 4.9±5.1 | 0.97 | 1 |
|  | Motor Aspects of Experience | 1.3±2.1 | 0.2±0.5 | 0.16 | 1 |
|  | Beck Anxiety Inventory | 7.5±7.6 | 3.6±4.6 | 0.048 | 0.96 |
|  | Beck Depression Inventory | 6.0±6.0 | 4.3±6.2 | 0.64 | 1 |
|  | Everyday Cognition | 13.0±2.6 | 12.2±0.5 | 0.93 | 1 |
| *National Institute of Health (NIH); Delis-Kaplan Executive Function System (DKEFS); Rey Auditory Verbal Learning Test (RAVLT).* | | | | | |

***Table S2. CSF PrP levels by mutation status.*** *Data from Figure 1 summarized in tabular form. CSF PrP data averaged for each participant and then grouped by mutation and presented as mean±sd across participants.*

| **mutation** | **CSF [PrP] ng/mL** | ***N* participants** |
| --- | --- | --- |
| none | 391.5 ± 106.7 | 11 |
| P102L | 274.0 ± 54.8 | 3 |
| D178N | 168.6 ± 37.6 | 5 |
| E200K | 291.8 ± 126.7 | 6 |
| other | 358.1 ± 175.8 | 4 |

***Table S3. Additional measures of cognitive, psychiatric, motor and daily functioning for one RT-QuIC positive MGH study participant.*** *Visits were separated by two months. Normative scores (based on normative data matched by age, gender, and education) are provided according to the published standard for each test.*

|  | | **Visit 1** | | **Visit 2** | | **Reliable**  **Change**  **Interval** |  |
| --- | --- | --- | --- | --- | --- | --- | --- |
| **Assess-ment scores** |  | Raw | Standard (%ile) | Raw | Standard (%ile) |  |  |
|  | Montreal Cognitive Assessment | 27 | -- | 25 | -- | +2.47 |  |
|  | NIH Toolbox Overall Cognition | 109 | -- | 104 | -- | +10.17 |  |
|  | Digital clock drawing test | 51 | -- | 56 | -- | +16.04 |  |
|  | Trailmaking test, Part A | 40 | 0.11 (54) | 51 | -0.67 (25) | +8.49* (↓) |  |
|  | Trailmaking test, Part B | 178 | -1.75 (4) | 120 | -0.45 (33) | +13.17* (↑) |  |
|  | DKEFS Color-word interference test, inhibition time | 63 | 12 (75) | 70 | 10 (50) | +12.80 |  |
|  | DKEFS Color-word interference test, switching time | 86 | 9 (37) | 73 | 11 (63) | +11.90* (↑) |  |
|  | DKEFS Letter Fluency | 43 | 12 (75) | 37 | 11 (63) | +16.11 |  |
|  | DKEFS Category Fluency | 38 | 12 (75) | 36 | 11 (63) | +14.27 |  |
|  | DKEFS Category Switching | 12 | 10 (50) | 14 | 11 (63) | +3.37 |  |
|  | Grooved pegboard test, dominant hand | 122 | -2.14 (2) | 131 | -2.60 (<1) | +11.42 |  |
|  | Grooved pegboard test, non-dominant hand | 157 | -2.67 (<1) | 155 | -2.59 (<1) | +13.59 |  |
|  | RAVLT– delayed recall | 6 | -0.42 (34) | 6 | -0.42 (34) | +3.20 |  |
| **Question-naire scores** | Epworth Sleepiness Scale | 3 | -- | 2 | -- | -- |  |
|  | Athens Insomnia Scale | 7 | -- | 1 | -- | -- |  |
|  | Motor Aspects of Experience | 1 | -- | 0 | -- | -- |  |
|  | Beck Anxiety Inventory | 9 | -- | 5 | -- | -- |  |
|  | Beck Depression Inventory | 4 | -- | 6 | -- | -- |  |
|  | Everyday Cognition | 12 | -- | 12 | -- | -- |  |
| *Delis-Kaplan Executive Function System (DKEFS); Rey Auditory Verbal Learning Test (RAVLT); Standard scores for DKEFS subtests are presented as scaled scores (M=10, SD=3); Standard scores for Trailmaking test, Grooved pegboard, and RAVLT are presented as z-scores (M=0, SD=1). The reliable change interval was calculated based on the standard deviation of the residuals predicting time 2 scores from time 1 scores (using a two-tailed 90% confidence interval) within the non-carrier control group. After raw scores were standardized based on published age and gender norms for each test, this individual performed within the average range for the majority of cognitive measures administered at both time 1 and time 2. The notable exception is the grooved pegboard test where this individual’s performance consistently fell within the impaired range for both dominant and non-dominant hands, and likely represents an area of relative weakness that is consistent over time. Although performance on Trailmaking Test Part B was also impaired at time point 1, performance significantly improved across the retest interval and the score was within the average range at time 2. *Tests falling outside of the confidence interval of reliable change between visits with accompanying arrows indicating whether performance at visit 2 was better* (↑) *or worse* (↓) *than performance at visit 1.* | | | | | | | |

# STROBE Checklist

|  | Item No | Recommendation | Page  No |
| --- | --- | --- | --- |
| **Title and abstract** | 1 | (*a*) Indicate the study’s design with a commonly used term in the title or the abstract | 1 |
|  |  | (*b*) Provide in the abstract an informative and balanced summary of what was done and what was found | 1 |
| Introduction | | | |
| Background/rationale | 2 | Explain the scientific background and rationale for the investigation being reported | 2 |
| Objectives | 3 | State specific objectives, including any prespecified hypotheses | 2, 13 |
| Methods | | | |
| Study design | 4 | Present key elements of study design early in the paper | 2 |
| Setting | 5 | Describe the setting, locations, and relevant dates, including periods of recruitment, exposure, follow-up, and data collection | 1 |
| Participants | 6 | (*a*) *Cohort study*—Give the eligibility criteria, and the sources and methods of selection of participants. Describe methods of follow-up  *Case-control study*—Give the eligibility criteria, and the sources and methods of case ascertainment and control selection. Give the rationale for the choice of cases and controls  *Cross-sectional study*—Give the eligibility criteria, and the sources and methods of selection of participants | 3,13 |
|  |  | (*b*) *Cohort study*—For matched studies, give matching criteria and number of exposed and unexposed  *Case-control study*—For matched studies, give matching criteria and the number of controls per case | 3 |
| Variables | 7 | Clearly define all outcomes, exposures, predictors, potential confounders, and effect modifiers. Give diagnostic criteria, if applicable | 3,4,5,7,8 |
| Data sources/ measurement | 8* | For each variable of interest, give sources of data and details of methods of assessment (measurement). Describe comparability of assessment methods if there is more than one group | 13-15 |
| Bias | 9 | Describe any efforts to address potential sources of bias | 11 |
| Study size | 10 | Explain how the study size was arrived at | 13 |
| Quantitative variables | 11 | Explain how quantitative variables were handled in the analyses. If applicable, describe which groupings were chosen and why | 3-8 |
| Statistical methods | 12 | (*a*) Describe all statistical methods, including those used to control for confounding | 3-8 |
|  |  | (*b*) Describe any methods used to examine subgroups and interactions | N/A |
|  |  | (*c*) Explain how missing data were addressed | 13 |
|  |  | (*d*) *Cohort study*—If applicable, explain how loss to follow-up was addressed  *Case-control study*—If applicable, explain how matching of cases and controls was addressed  *Cross-sectional study*—If applicable, describe analytical methods taking account of sampling strategy | 13 |
|  |  | (*e*) Describe any sensitivity analyses | N/A |

| Results | | | |
| --- | --- | --- | --- |
| Participants | 13* | (a) Report numbers of individuals at each stage of study—eg numbers potentially eligible, examined for eligibility, confirmed eligible, included in the study, completing follow-up, and analysed | 3,13 |
|  |  | (b) Give reasons for non-participation at each stage | 13 |
|  |  | (c) Consider use of a flow diagram |  |
| Descriptive data | 14* | (a) Give characteristics of study participants (eg demographic, clinical, social) and information on exposures and potential confounders | 3 |
|  |  | (b) Indicate number of participants with missing data for each variable of interest | 3-8 |
|  |  | (c) *Cohort study*—Summarise follow-up time (eg, average and total amount) | 1,3 |
| Outcome data | 15* | *Cohort study*—Report numbers of outcome events or summary measures over time | N/A |
|  |  | *Case-control study—*Report numbers in each exposure category, or summary measures of exposure |  |
|  |  | *Cross-sectional study—*Report numbers of outcome events or summary measures |  |
| Main results | 16 | (*a*) Give unadjusted estimates and, if applicable, confounder-adjusted estimates and their precision (eg, 95% confidence interval). Make clear which confounders were adjusted for and why they were included | N/A |
|  |  | (*b*) Report category boundaries when continuous variables were categorized |  |
|  |  | (*c*) If relevant, consider translating estimates of relative risk into absolute risk for a meaningful time period |  |
| Other analyses | 17 | Report other analyses done—eg analyses of subgroups and interactions, and sensitivity analyses | N/A |
| Discussion | | | |
| Key results | 18 | Summarise key results with reference to study objectives | 3-8 |
| Limitations | 19 | Discuss limitations of the study, taking into account sources of potential bias or imprecision. Discuss both direction and magnitude of any potential bias | 12 |
| Interpretation | 20 | Give a cautious overall interpretation of results considering objectives, limitations, multiplicity of analyses, results from similar studies, and other relevant evidence | 11-13 |
| Generalisability | 21 | Discuss the generalisability (external validity) of the study results | 12 |
| Other information | | | |
| Funding | 22 | Give the source of funding and the role of the funders for the present study and, if applicable, for the original study on which the present article is based | 17 |
